# Ulnar nerve integrity predicts 1-year outcome in cervical spinal cord injury

**DOI:** 10.1186/s42466-019-0017-1

**Published:** 2019-05-22

**Authors:** Andreas Hug, Christian Schuld, Bettina Mürle, Markus Böttinger, EMSCI study group, Norbert Weidner, Rüdiger Rupp

**Affiliations:** 1 Spinal cord injury center, Heidelberg University Hospital, Schlierbacher Landstraße 200a, 69118 Heidelberg, Germany; 2 Department of Neuroradiology, University Medical Center Mannheim, Medical Faculty Mannheim, Heidelberg University, 68167 Mannheim, Germany

**Keywords:** Spinal cord injury, Electroneurography, Ulnar nerve, Prognosis, Outcome

## Abstract

**Background:**

Accurate predictors of neurological recovery after cervical spinal cord injury are needed. Particularly, to tailor adequate rehabilitation plans. However, objective and quantifiable predictors are sparse.

**Methods:**

Within the prospective European Multicenter Study about Spinal Cord Injury (EMSCI) registry, cervical spinal cord injury patients are monitored at fixed follow up visits (2, 4, 12, 24, and 48 weeks after injury) clinically and with ulnar nerve electroneurography. Associations of ulnar nerve compound muscle action potential amplitudes (CMAP) with American Spinal Cord Injury Association (ASIA) impairment scale (AIS) grades over time were analyzed using linear mixed modeling. Applying logistic regression, the prognostic value of within 4-week ulnar nerve CMAP for 1-year AIS was analyzed. To account for missing data, (1) last observation carried forward and (2) multiple imputation methods were applied. For model derivation, our centers’ cohort (EMSCI-HD) was analyzed. For model validation the cohort of other centers (EMSCI-nonHD) was used.

**Results:**

In the EMSCI-HD cohort, the median age (interquartile range (IQR)) was 52 (34–67) years. 58% were male. The initial AIS distribution was: A = 31%, B = 17%, C = 30%, and D = 22%). In the EMSCI-nonHD cohort, the median age was 49 (32–65) years. Compared to the EMSCI-HD cohort more patients were male (79%, *p* = 0.0034). The AIS distribution was: A = 33%, B = 13%, C = 21%, and D = 33%).

In complete-case mixed model analyses (EMSCI-HD: *n* = 114; EMSCI-nonHD: *n* = 508) higher ulnar nerve CMAP were associated with better AIS grades over the entire follow up period. In complete-case logistic regression (EMSCI-HD: *n* = 90; EMSCI-nonHD: *n* = 444) higher ulnar nerve CMAP was an independent predictor of better AIS grades. The odds ratio for within 4-week ulnar nerve CMAP to predict 1-year AIS grade D versus A-C in the EMSCI-HD cohort was 1.24 per millivolt (confidence interval 1.07–1.44). The model was validated in an independent cervical spinal cord injury (EMSCI-nonHD) cohort (odds ratio 1.09, confidence interval 1.03–1.17).

**Conclusions:**

In cervical spinal cord injury, the consideration of early ulnar nerve CMAP improves prognostic accuracy, which is of particular importance in patients with clinical grading uncertainties.

**Electronic supplementary material:**

The online version of this article (10.1186/s42466-019-0017-1) contains supplementary material, which is available to authorized users.

## Background

Early and accurate prediction of neurological recovery is a major goal in the care of traumatic cervical spinal cord injury (SCI) for (1) providing patients with a realistic prognosis, (2) an effective and tailored rehabilitation plan and (3) the correct risk stratification in clinical trials. One very robust predictor of neurological recovery is the baseline American Spinal Injury association impairment scale (AIS) grade [12, 22, 26]. For traumatic cervical SCI there are accurate model systems to predict AIS conversions on the basis of the initial AIS grade over a time period of 1 year [10]. Except for AIS D and E patients (no room for improvement due to a ceiling effect of the scale), patients with more severe injuries at baseline are generally less likely to convert to better AIS grades [22]. However, early on AIS grading is quite unreliable/inaccurate due to (1) patient specific issues (e. g. deteriorated conscious state) [2, 11, 16, 24] or (2) uncertainties of investigators in applying the correct AIS grade [20, 21]. Not only but in particular for these situations, an objective and quantifiable parameter would help to improve prognostic accuracy. Except for sensory and motor evoked potentials - which often are not simple to apply and interpret in the acute setting - no such parameter is currently available [4–7, 17]. Moreover, independent predictors, which improve prognostic accuracy of clinical grading (AIS) alone, were not extensively analyzed.

Electroneurography (ENG) of the ulnar nerve is a standard clinical technique, which is widely available, robust and mobile. The technique enables examiners to make reliable measurements even under intensive care conditions where patients with cervical SCI usually stay for the first days to weeks after injury. Furthermore, resulting parameters like compound muscle action potential (CMAP) amplitudes or nerve conduction velocities (NCV) are easy to quantify. So far, one study demonstrated that pathologic ulnar nerve ENG (i.e. reduced CMAP amplitudes) is associated with worse hand function in cervical SCI. Motoneuron damage (motor neuronopathy) within the anterior horn of the cervical spinal cord was hypothesized as the underlying mechanism. Except for worse hand function [3] and the possibility to discern between a central versus peripheral paresis pattern [6], ulnar nerve ENG was not systematically analyzed in cervical SCI as predictor of neurological recovery (AIS conversion) and its role as a surrogate marker for axonal injury to corticospinal neurons within the cervical spinal cord.

Using the Heidelberg cohort of the European multicenter study about spinal cord injury (EMSCI) database (http://www.emsci.org) as a derivation data set, we could demonstrate that increased AIS completeness is associated with decreased ulnar nerve CMAP amplitudes. Moreover, we could demonstrate that early on (within 4 weeks after injury) preserved ulnar nerve CMAP amplitudes are an independent predictor of good neurological recovery at 1 year after injury (AIS D versus A-C). Finally, we could validate our prediction model using the total non-HD EMSCI dataset demonstrating robustness/inferability to other SCI centers.

## Methods

### EMSCI database

The EMSCI database is a prospective registry of acute SCI patients (http://www.emsci.org; ClinicalTrials.gov Identifier: NCT01571531). In this registry, the recovery of patients is followed up for a total of 1 year after the incident traumatic or ischemic SCI in order to provide the clinical basis for new interventional therapies [8]. Assessments take place at fixed time points after the incident injury: very acute (within 2 weeks), acute I (4 weeks), acute II (12 weeks), acute III (24 weeks), and chronic (48 weeks). At each visit patients are graded clinically according to the AIS (part of the International Standards for Neurological Classification of Spinal Cord Injury (ISNCSI); Additional file 1: Table S1) [13, 15]. Eleven of 21 centers within the EMSCI-project additionally collect ulnar nerve ENG data in the cervical SCI population. The ENG recordings are standardized between the 11 participating EMSCI-centers with respect to the montage of electrodes and stimulation parameters. The study was approved by the local institutional review boards/independent ethics committees of each participating center. Informed consent was obtained before any study related procedure.

### Data analysis and statistical analysis

For the derivation data set the EMSCI patients from Heidelberg were used (EMSCI-HD). Of those, only patients with a cervical neurological level of injury (NLI) were included (segments C1 to T1; *n* = 249). Furthermore, only patients with at least one clinical AIS grading and one ulnar nerve CMAP measurement were included in the final derivation data set (*n* = 114). A linear repeated measures mixed model was fit to the data with ulnar nerve CMAP amplitude as a continuous dependent variable (SAS®, PROC MIXED). As independent fixed effects variables, the following categorical factors were used: AIS (four levels: A to D), NLI (nine levels: C1-T1), visits (four levels: very acute, acute I, acute II, acute III, chronic), the interaction effect between AIS and NLI, and the interaction effect between AIS and visit. Moreover, age was entered into the final model as continuous variable. The model allowed a random intercept for each patient with an unstructured covariance structure grouped by AIS. Follow up visits were entered as a repeated measures effect into the final regression model with a first order autoregressive covariance structure. For hypothesis generation, the model was first fit for the right ulnar nerves. Hypothesis tests were then performed on statistically significant effects (alpha level < 0.05) using the same model and ulnar CMAP values of left hands. Alpha levels of < 0.05 were regarded as statistically significant.

As additional control for missing data we applied multiple imputation (*n* = 10) of the variables (ulnar CMAP, AIS) assuming a missing at random data structure and a non-monotone missingness pattern. Hence, a chained equation method with full imputation of missing data was applied (regression for CMAP and regression or ordinal logistic regression for AIS). Mixed model analysis was then performed by each single imputation and overall fitted means were calculated (SAS®, PROC MI and PROC MIANALYZE; STATA® ICE) [9, 19]. In case, all CMAP measurements were missing, no imputations were made (i.e. the observation was deleted).

Once higher CMAP amplitudes were identified to be associated with better neurological recovery, we designed a logistic multivariable model in order to analyze whether early ulnar CMAP amplitudes are an independent predictor of 1-year neurological recovery (SAS®, PROC LOGISTIC). For this purpose, we defined the first 4 weeks after injury as the prediction time frame (visits very acute and acute I). Primarily, we used the very acute values as predictors. Only if the very acute visit values were missing (AIS or CMAP or NLI), we used the acute I values. For the 1-year endpoint we used a last observation carried forward approach (complete-case analysis set). The outcome variable was dichotomized into AIS A-C versus AIS D (with D indicating a “good”/“useful” neurological outcome). The initial AIS grade was entered into the model as categorical factor dichotomized into motor complete (A and B) versus motor incomplete (C and D). In the initial statistical analysis plan, ordinal scaling (A = 0, B = 1, C = 2, D = 3) was favored. However, all initial AIS D patients in the EMSCI-HD cohort were still AIS D at the final follow up visit (ceiling effect of the AIS). Hence, a separate category for AIS D would have been uninformative due to a perfect prediction of the final AIS (D versus A-C). The CMAP amplitude was entered into the model as continuous variable. Furthermore, NLI and age were entered in the first step of the model. Using a backward variable elimination approach (alpha level of < 0.05 to stay in the model) the following variables were retained in the final model: initial AIS and CMAP amplitude. First, we analyzed the logistic model for the right hand. For hypothesis generation alpha levels of < 0.05 were regarded as statistically significant. For hypothesis testing, the significant effects were then reanalyzed for the left hand using the same model. Alpha levels of < 0.05 were regarded statistically significant. ROC curves were generated and compared via a bootstrap approach and plotted for each independent variable separately (pROC, R) [18]. Predicted probabilities of the full logistic model were used to plot the final model ROC curve. For differences in ROC curve areas under the curve (AUC) an alpha level < 0.05 was regarded statistically significant.

Due to missing data we also investigated the results of the logistic model with the use of multiple imputation. In case all CMAP measurements were missing, no imputations were made (i.e. observation was deleted). After full imputation *n* = 120 patients contributed to the model. A total of 10 data sets were imputed and afterwards an overall mean of the estimates with corresponding 95%-confidence intervals were calculated (SAS®, PROC MI and PROC MIANALYZE).

For validation of the logistic prediction model, the EMSCI data set of other centers was used (EMSCI-nonHD). Data management was equivalent to the EMSCI-HD dataset. Due to side symmetrical results for right and left ulnar nerves, data are presented only for the right ulnar nerves.

Data management and statistical analysis was performed using SAS® version 9.2, Stata® version 11 and R version 2.15.2.

## Results

### Derivation data set. Demographic and clinical data

**Fig. 1:**
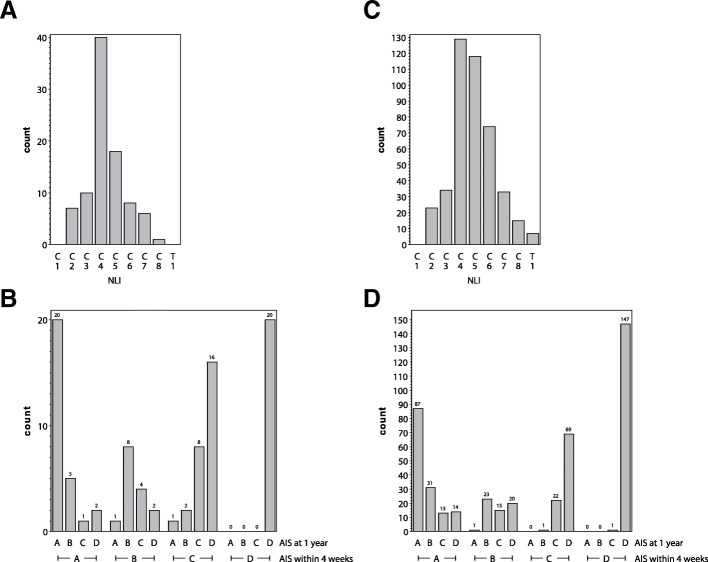
**a** NLI distribution (**b**) AIS conversions of the EMSCI-HD cohort. **c** NLI distribution (**d**) AIS conversions of the EMSCI-nonHD cohort. Complete-case analysis: EMSCI-HD *n* = 90; EMSCI-nonHD *n* = 444

Over a recruiting period of 11 years (2001–2012) we identified 249 patients with cervical SCI (NLI C1-T1) in the EMSCI-HD cohort. Of those, 114 patients had sufficient data (at least one AIS grade and one ulnar CMAP measurement) for mixed model analysis. For logistic regression, 90 patients had sufficient data for complete-case statistical analysis (i.e., one AIS grading and one CMAP measurement within 4 weeks; last observation carried forward) in the EMSCI-HD cohort. Using multiple imputation, observations of 120 patients were informative for statistical analysis. For the complete-case data set age (median, interquartile range (IQR)) was 52 (34–67) years. Male to female ratio was 58/32 (Baseline clinical characteristics are given in Table 1). The distribution of the NLI revealed an expected maximum at the cervical levels C4 and C5 (Fig. 1a). The AIS conversion rates showed the typical pattern with more complete patients (AIS A) converting less likely to better AIS grades than less complete patients (AIS B, C; ceiling effect of the scale for AIS D) (Fig. 1b).

**Table 1:** Complete-case data set

	EMSCI-HD	EMSCI-nonHD	*p* value
	*N* = 90	*n* = 444	
Age (median years, IQR)	52 (34–67)	49 (32–65)	0.1678
Gender (count male, %)	58 (64.44)	350 (78.83)	**0.0034**
AIS initial (count, %)			0.0897
A	28 (31.11)	145 (32.66)	
B	15 (16.67)	59 (13.29)	
C	27 (30.00)	92 (20.72)	
D	20 (22.22)	148 (33.33)	
AIS end (count, %)			0.2301
A	22 (24.44)	88 (19.82)	
B	15 (16.67)	55 (12.39)	
C	13 (14.44)	51 (11.49)	
D	40 (44.44)	250 (56.31)	
Timing of visits (median weeks, IQR)			
Very acute	1.21 (0.86–1.71)	1.0 (0.43–1.57)	0.5871
Acute I	4.07 (3.57–4.64)	4.14 (3.57–4.71)	0.8436
Acute II	12.0 (11.29–12.29)	12.0 (11.57–12.71)	0.4226
Acute III	23.93 (22.86–24.29)	24.0 (23.29–25.0)	0.4665
Chronic	50.86 (45.0–56.86)	48.42 (46.92–52.29)	**< 0.0001**

*p*<0.05 values are typed bold

### Derivation data set. Mixed model analysis of ulnar nerve CMAP association with AIS grade over time

**Fig. 2:**
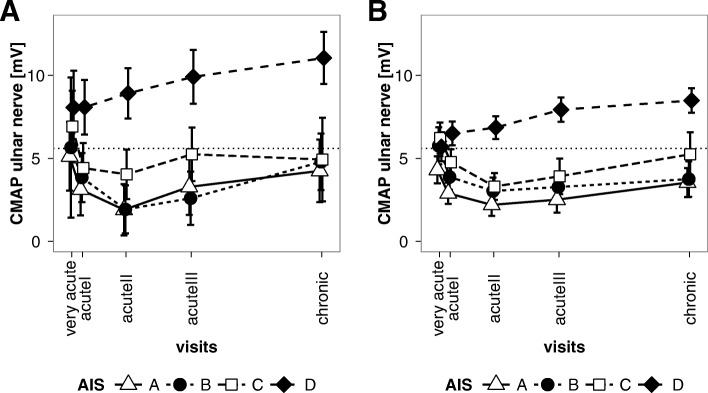
**a** Mixed model estimates of means and 95% confidence intervals of right ulnar CMAP amplitudes in the EMSCI-HD cohort over the 1-year follow up. **b** Mixed model estimates of means and 95% confidence intervals of right ulnar CMAP amplitudes in the EMSCI-nonHD cohort over the 1-year follow up

The ulnar nerve CMAP amplitude lower limit of normal in our neurophysiology laboratory is 5.6 mV. With respect to this limit, patients with AIS A-C exhibited borderline or decreased CMAP amplitudes over the total follow up period of 1 year. In contrast, patients with AIS D revealed CMAP amplitudes within normal limits over the entire follow up period (Fig. 2a).

In contrast to AIS D patients AIS A-C patients developed a drop of CMAP amplitudes from very acute to acute I and II time points (Fig. 2a).

### Derivation data set. Logistic model using initial AIS/CMAP to predict functional outcome at 1 year

**Fig. 3:**
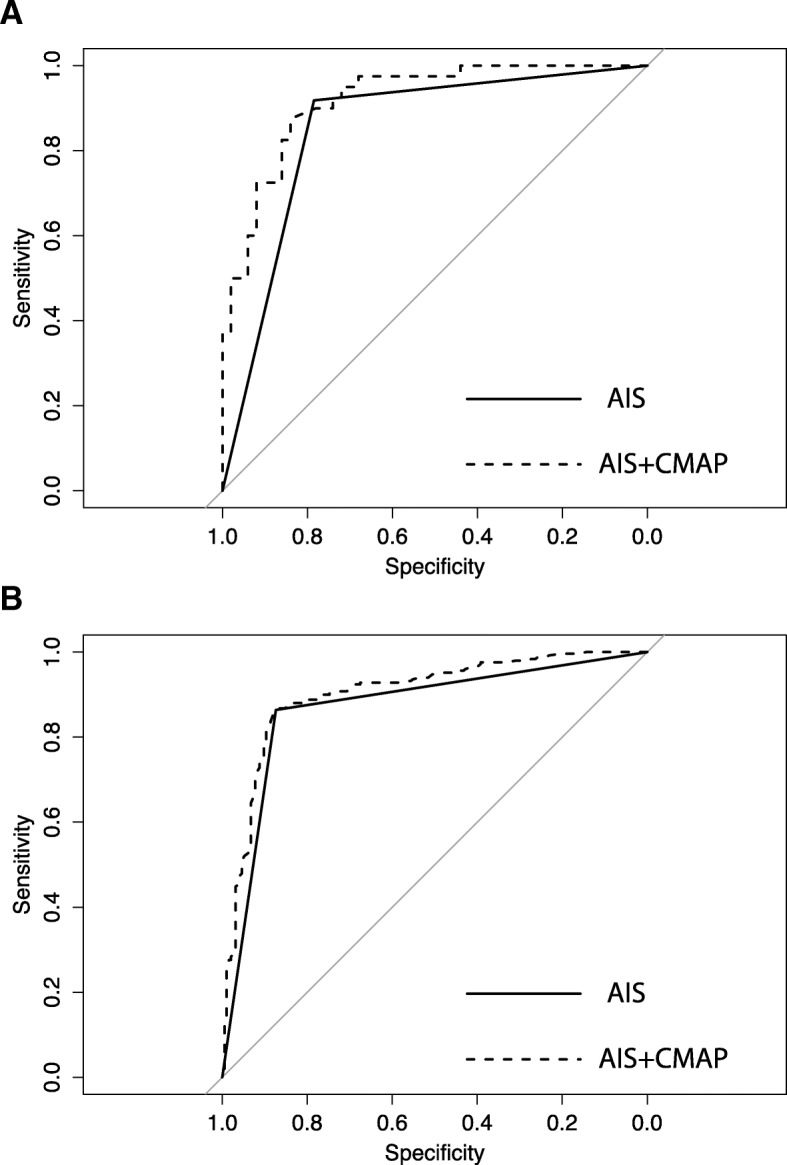
ROC curve analysis of logistic regression results. The solid line represents the logistic fit of the initial AIS (within first 4 weeks) for the prediction of good recovery AIS D versus A-C. The dashed line represents the logistic model fit of initial AIS + initial right ulnar nerve CMAP amplitude. The improved prediction accuracy of the full model (initial AIS + initial CMAP) versus initial AIS alone is significant in the EMSCI-HD (**a**) and EMSCI-nonHD (**b**) cohorts, respectively (*p* = 0.0017 and 0.0003). Complete-case analysis. (EMSCI-HD cohort *n* = 90; EMSCI-nonHD cohort *n* = 444)

Sufficient data for the complete-case statistical analysis was available in *n* = 90 patients. As expected, the main predictor of good outcome in multivariable logistic model was the initial AIS grade (dichotomized C-D versus A-B) with an OR of 37.61 (CI 9.49–149.03, *p* < 0.0001). Furthermore, logistic model analysis revealed that higher initial CMAP amplitudes were an independent predictor of good 1-year outcome (AIS D versus A-C). The OR per mV increase in CMAP amplitude was 1.24 (CI 1.07–1.44, *p* = 0.0025). The combination of AIS and CMAP amplitude performed better with respect to prediction accuracy than initial AIS alone as demonstrated by ROC curve analysis (Fig. 3a; for CMAP amplitude alone see Additional file 1: Figure S1). The difference between AUC of the AIS model (0.85; CI 0.78–0.92) compared to the AIS + CMAP model (0.92; CI 0.87–0.97) was statistically significant (*p* = 0.0017). Sensitivity and Specificity of the AIS + CMAP model for the prediction of 1-year outcome was 0.88 and 0.84, respectively. After multiple imputation a total of 120 patients were available for analysis. Results were similar to the complete-case analysis of the unimputed data set: OR 1.24, CI 1.03–1.48 (Additional file 1: Table S2).

### Validation data set

Demographic data was similar in the EMSCI-HD and EMSCI-nonHD data sets except for a slight preponderance of male patients in the EMSCI-nonHD cohort (Table 1). NLI distributions and AIS-conversions rates of the EMSCI-nonHD cohort are given in Fig. 1c and d. Re-analysis of the mixed model in the EMSCI-nonHD data set (*n* = 508) revealed similar results as for the EMSCI-HD cohort (Fig. 2b). Using the complete-case data set a total of 444 cases were informative for logistic regression analysis. Using the same logistic model as for the EMSCI-HD cohort, re-analysis in the EMSCI-nonHD cohort also revealed that baseline ulnar CMAP predicted good outcome (AIS D versus A-C) with an OR of 1.09 per mV CMAP amplitude increase (CI 1.03–1.17, *p* = 0.006). Compared to AIS alone the combination of AIS + CMAP revealed an improved prediction accuracy as demonstrated by ROC curve analysis (0.87 CI 0.84–0.9 versus 0.90 CI 0.87–0.93; *p* = 0.0003 for AUC difference; Fig. 3b). After multiple imputation (*n* = 10 imputations) 508 patients were informative for statistical analysis. Again, baseline ulnar CMAP was predictive for good outcome (AIS D versus A-C) with an OR of 1.10 per mV CMAP amplitude increase (CI 1.04–1.18, *p* = 0.0022). For logistic regression results see Additional file 1: Table S3. AIS-conversion rates for the complete-case and multiple imputation datasets are visualized in Additional file 1: Figure S2.

## Discussion

For patients with acute cervical SCI we could demonstrate (1) a positive correlation of ulnar nerve CMAP amplitudes with AIS incompleteness over time. (2) Within 4-week ulnar nerve axonal integrity is an independent predictor of good 1-year outcome. (3) The prediction model consisting of baseline AIS and ulnar nerve CMAP amplitude is robust and inferable to other cervical SCI cohorts.

This is the first clinical study, which demonstrates that a readily measurable and quantifiable damage to the peripheral nervous system (ulnar nerve CMAP measurements) is a useful surrogate marker for central nervous system recovery (AIS grade) after cervical SCI. The underlying pathomechanism of reduced ulnar nerve CMAP amplitudes after acute cervical SCI remains only incompletely understood. The identified time course with an amplitude nadir not at the very acute (2 weeks) but later time points argues for an ongoing neuronal/axonal degeneration (e.g. Wallerian degeneration of lower motoneurons). Data supporting this theory come from one study where it was shown that lower ulnar nerve CMAP amplitudes in patients with low cervical NLI (C8/T1) are associated with worse hand function. There, it was hypothesized, that reduced ulnar CMAP amplitudes may be due to lower motoneuron damage [3]. To decipher the underlying pathomechanism in clinical SCI, further investigations are warranted. For this purpose, very early screening (day 1) with daily monitoring of Wallerian degeneration would be necessary. Possibly, the difference between baseline ulnar nerve CMAP and the CMAP after Wallerian degeneration might be an even better marker for lower motoneuron damage.

Regardless of the underlying mechanism for reduced ulnar CMAP amplitudes, they also seem to reflect axonal damage to upper (corticospinal) motoneurons since increased AIS completeness and not the NLI was the main predictor for low CMAP amplitudes in our analyses. A recent MRI study supports this idea, with more AIS complete injuries associated with an increased intramedullary length of lesion [1]. Formally it remains to be established, if decreased ulnar CMAP amplitudes are a mere indicator for ventral horn (lower motoneuron) damage, or could also serve as a surrogate marker for axonal damage of corticospinal motoneurons. The fact of an association with neurological recovery (measured by AIS grade) in our study, argues for the suitability as such a surrogate marker. The most accurate cut-off value of ulnar nerve CMAP amplitudes for the prediction of neurological outcome was not explicitly addressed in this study. However, the lower limit of ulnar nerve CMAP amplitude at our site (5.6 mV) seems to be a suitable value as demonstrated by our mixed model analysis.

Except for accurate early prognosis and planning of rehabilitation interventions, early on measurements of ulnar nerve CMAP amplitudes could help to improve risk stratification in randomized clinical trials of SCI. Such accurate stratification is of utmost importance in acute SCI trials to balance baseline variables since slow recruitment is a major problem and treatment effects of novel experimental drugs are usually small [10, 14, 23, 25].

A further advantage of ulnar nerve CMAP measurements is the easy and objective application even in the intensive care setting, where patients usually are only partly cooperative [2, 11, 16, 24]. Furthermore, subjective errors due to uncertainties of the correct AIS application [20, 21] could be partly compensated by the added prognostic value of CMAP measurements.

Our analysis has several limitations. First of all, the data contain many missing values. We addressed this problem by different methods, i.e. linear mixed model, last observation carried forward and multiple full imputation analysis. Due to the robustness of the detected effects across the different analysis sets, we are confident that the CMAP effect truly exists. Furthermore, our analysis was based on the so far largest sample size with regards to the prognostic significance of ulnar nerve CMAP amplitudes. A further weakness is the possibility of a systematic bias regarding missing CMAP measurements in more severe SCI patients. Analyses of missing case patterns however, suggested a missing at random data pattern. Due to the study design, inference is limited to the spinal segments C8-T1 (ulnar nerve). Considering the more extensive range of spinal segments, CMAP amplitudes of median nerves (C5-T1) might be more accurate surrogate predictors of neurological outcome. Due to the time course of Wallerian degeneration, electroneurography will probably not be a useful prognostic predictor for ultra-early stages of SCI (i.e., hours after injury). For these ultra-early time windows where clinical AIS grading becomes more and more inaccurate, other surrogate markers to decipher spinal shock with and without permanent spinal cord injury are urgently needed for surgical/medical decision-making [27]. Our data is representative for Mid-European SCI patients and care facilities. Inferability to other parts of the world needs to be demonstrated.

## Conclusions

The consideration of early ulnar nerve CMAP amplitudes improves prognostic accuracy of 1-year neurologic outcomes in cervical SCI patients. This could be of particular importance in patients who are difficult to grade clinically. Moreover, improved accuracy could prove significant for proper risk stratification in clinical trials.

## Additional file

Additional file 1: Supplementary appendix. (DOCX 379 kb)

## Abbreviations

AIS: ASIA-Impairment Scale
ASIA: American Spinal Injury Association
AUC: Area under the Curve
C: Cervical
CI: Confidence Interval
CMAP: Compound muscle action potential
EMSCI: European Study about Spinal Cord Injury
ENG: Electroneurography
HD: Heidelberg Center
IQR: Interquartile Range
ISNCSCI: International Standards for the Neurological Classification of Spinal Cord Injury
mV: Milli-Volt
NCV: Nerve Conduction Velocity
NLI: Neurological Level of Injury
OR: Odds Ratio
ROC: Receiver Operator Characteristics
SCI: Spinal Cord Injury
T: Thoracic
