# Supplementary material for: Ulnar nerve integrity predicts 1-year outcome in cervical spinal cord injury
Source: Neurol Res Pract. 2019 May 22;1:11. doi: 10.1186/s42466-019-0017-1 (PMC7650063; doi:10.1186/s42466-019-0017-1)
Supplement: Supplementary file 1 — Supplementary appendix. (DOCX 379 kb) [file 42466_2019_17_MOESM1_ESM.docx]

Suppl. Methods

*Neurophysiology.* Electroneurography was done using a standard protocol. All recordings were performed on a Schwarzer topas 4-channel EMG system (Natus® neurology, Munich, Germany) or a Nicolet Viking III P by two experienced technicians. The nerves were stimulated electrically with a bipolar stimulation block (distance anode-cathode one centimeter) with the cathode distally. Compound muscle action potentials (CMAP) were recorded using 10 millimeter diameter silver-silver chloride skin surface electrodes with a tendon-belly montage. The ulnar nerve was stimulated at two different sites (wrist and elbow) and CMAPs were registered from the abductor digiti minimi muscle (ADM). Amplitudes of the CMAPs were recorded in milli-Volt (mV).

Table S1

| Grade A | No motor or sensory function is preserved in the sacral  segments S4–S5 |
| --- | --- |
| Grade B | Sensory but not motor function is preserved below the  neurological level and includes the sacral segments S4–S5 |
| Grade C | Motor function is preserved below the neurological level, and  more than half of key muscles below the neurological level  have a muscle grade of less than 3 |
| Grade D | Motor function is preserved below the neurological level, and  at least half of key muscles below the neurological level have  a muscle grade of 3 or more |
| Grade E | Motor and sensory function are normal |

American Spinal Injury Association/International Spinal Cord Society neurological standard scale

Table S2

| Parm | Odds Ratio | LCLMean | UCLMean | p value |
| --- | --- | --- | --- | --- |
| Intercept | 0.71 | 0.18 | 2.74 | 0.616 |
| AIS (C-D vs A-B) | 29.98 | 7.52 | 119.50 | <.0001 |
| CMAP (per mV) | 1.24 | 1.03 | 1.48 | 0.021 |

Mean of 10 imputations

Table S3

| Parm | Odds Ratio | LCLMean | UCLMean | p value |
| --- | --- | --- | --- | --- |
| Intercept | 1.21 | 0.73 | 2.00 | 0.452 |
| AIS (C-D vs A-B) | 30.65 | 16.29 | 57.65 | <.0001 |
| CMAP (per mV) | 1.10 | 1.04 | 1.18 | 0.002 |

Mean of 10 imputations

Figure S1


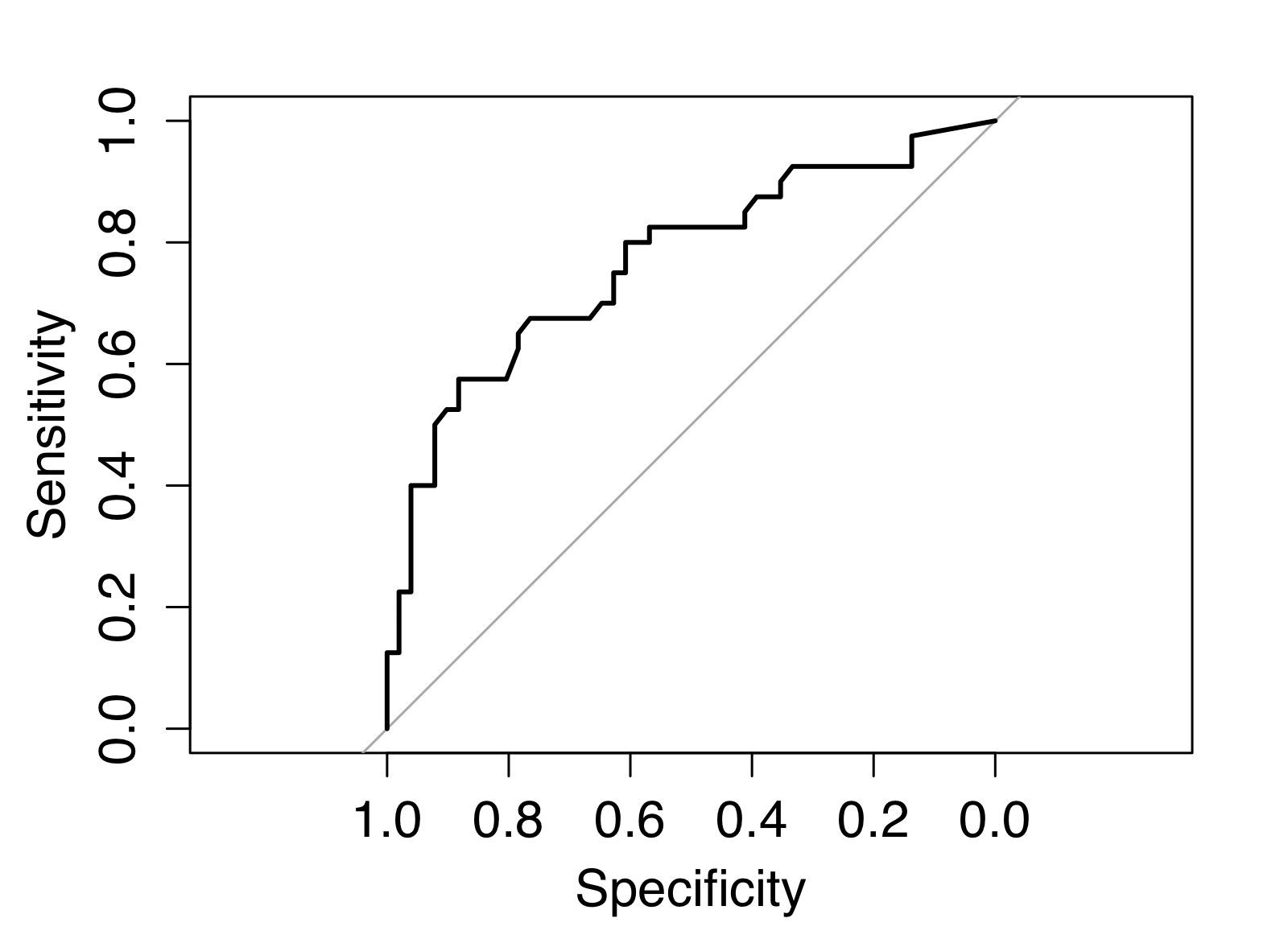


ROC curve analysis of within 4-week ulnar nerve CMAP amplitudes as continuous variable predicting 1-year AIS D versus AIS A-C in the EMSCI-HD cohort.

Figure S2


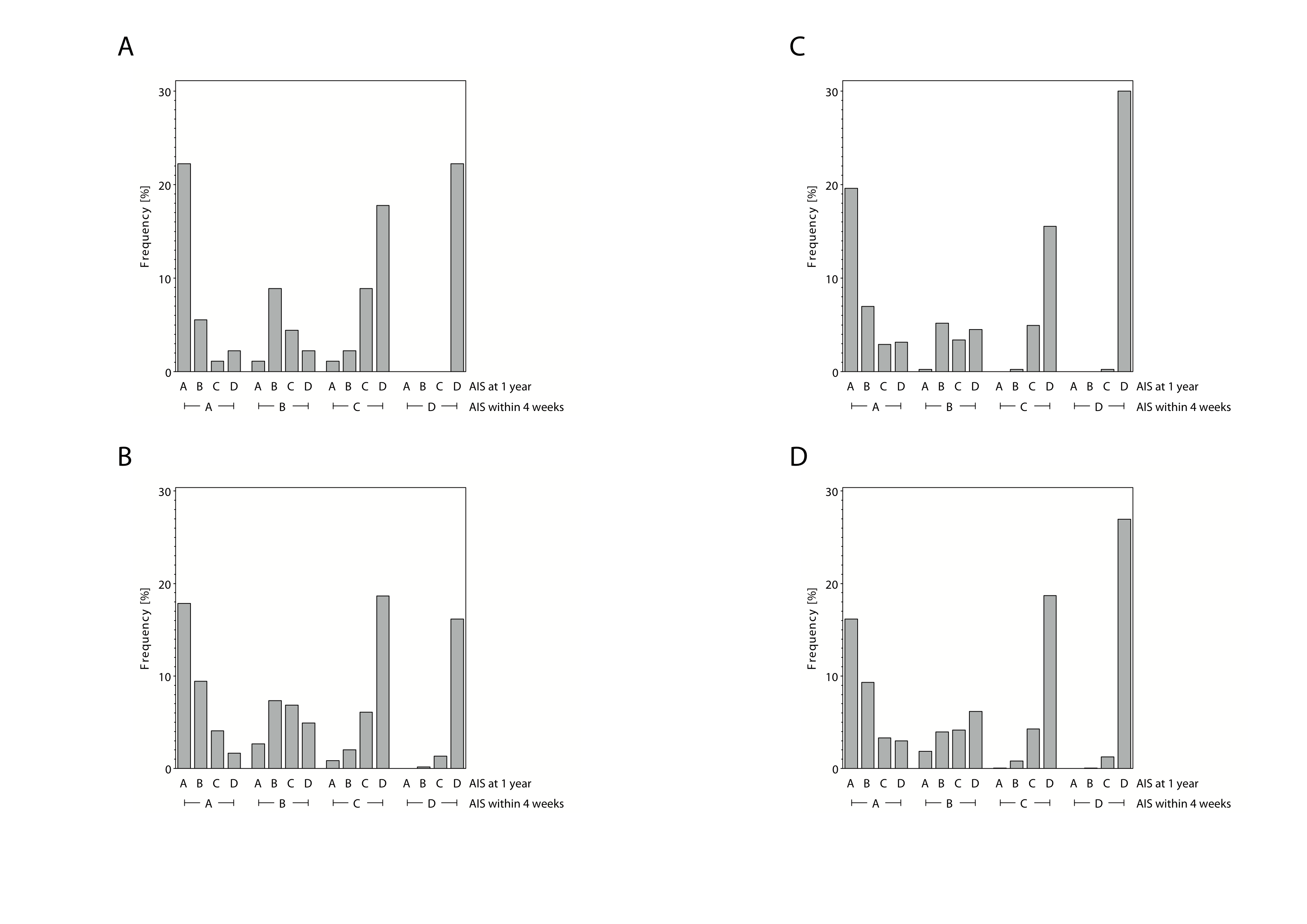


Relative (%) AIS conversion rates. (A) complete-case EMSCI-HD (B) multiple imputation (10 imputations) EMSCI-HD (C) complete-case EMSCI-nonHD (D) multiple imputation (10 imputations) EMSCI-nonHD
